# Effect of Commercial Children’s Mouthrinses and Toothpastes on the Viability of Neonatal Human Melanocytes: An In Vitro Study

**DOI:** 10.3390/dj11120287

**Published:** 2023-12-12

**Authors:** Shilpi Goenka, Hsi-Ming Lee

**Affiliations:** 1 Department of Biochemistry and Cell Biology, Stony Brook University, Stony Brook, NY 11794, USA; 2 Department of Biomedical Engineering, Stony Brook University, Stony Brook, NY 11794, USA; 3 Department of Oral Biology and Pathology, School of Dental Medicine, Stony Brook University, Stony Brook, NY 11794, USA; hsi-ming.lee@stonybrookmedicine.edu

**Keywords:** children’s toothpastes, children’s mouthrinses, gingiva, primary human melanocytes, cytotoxicity, sodium lauryl sulfate, cetylpyridinium chloride

## Abstract

In this study, we examined the cytotoxic effects of six commercial children’s mouthrinses (designated as #1, #2, #3, #4, #5, and #6) and four commercial children’s toothpastes (designated as #1, #2, #3, and #4) on primary human neonatal melanocytes that were used as a representative model for oral melanocytes. Mouthrinses diluted directly with culture medium (1:2, 1:5, 1:10, 1:100, and 1:1000) were added to monolayers of melanocytes for 2 min, followed by 24 h recovery, after which MTS cytotoxicity assay was conducted. The extracts of each toothpaste were prepared (50% *w*/*v*), diluted in culture medium (1:2, 1:5, 1:10, 1:50, 1:100, and 1:1000), and added to cell monolayers for 2 min (standard brushing time), followed by an analysis of cell viability after 24 h. Results showed that all mouthrinses except mouthrinse #4 showed significantly greater loss of cell viability, ascribed to cetylpyridinium chloride (CPC) that induced significant cytotoxicity to melanocytes (IC_50_ = 54.33 µM). In the case of toothpastes, the examination of cellular morphology showed that a 2 min exposure to all toothpaste extracts induced a concentration-dependent decline in cell viability, pronounced in toothpaste containing sodium lauryl sulfate (SLS) detergent. Further results suggested SLS to be the critical driver of cytotoxicity (IC_50_ = 317.73 µM). It is noteworthy that toothpaste #1 exhibited much lower levels of cytotoxicity compared to the other three toothpastes containing SLS. Taken together, these findings suggest that the melanocytotoxicity of children’s mouthrinse (#4) and toothpaste (#1) is comparatively low. To the best of our knowledge, this is the first study to examine the impact of children’s toothpastes and mouthrinses on neonatal primary human melanocytes. Future studies to investigate these findings in a realistic scenario replicating oral cavity conditions of the presence of microbiota, pellicle layer and saliva, and other cell types are warranted.

## 1. Introduction

Children are one of the groups that are particularly susceptible to the development of caries, which is a preventable condition [1]. The incidence of caries in permanent teeth was found to be three times higher among children who had previously experienced caries in their primary teeth [2]. Early childhood caries has an incidence of 48%, which has remained constant over the last three decades [3]. Toothpastes are oral care products used in conjunction with toothbrushes to maintain gingival health and prevent caries, plaque, and tooth decay [4,5,6]. Even though mechanical cleaning by the use of toothpastes with toothbrushes is essential for preventing oral pathologies, some oral biofilms, such as those found in buccal pits, fissures, gingival edges, and posterior interproximal regions, where most oral diseases are likely to develop, are practically hard to eradicate entirely [7,8]. Consequently, the use of chemical rinses (mouthwashes or mouthrinses), another popular oral hygiene product, can be effective in oral hygiene in conjunction with mechanical approaches. In addition, mouthrinses offer a better alternative and are less tedious compared to manual cleaning (that is accomplished by toothpastes used with manual or electric toothbrushes) in special needs children populations with cognitive or motor disabilities [9,10]. Moreover, mouthrinses are commonly used as a pre-procedural rinse or after surgery. The use of mouthrinses has seen an upsurge, in part, due to its capacity to reduce the severity of COVID-19 through its rapid virucidal activity against the SARS-CoV-2 virus [11,12,13]. Mouthrinses that are marketed for use by children are made appealing by the incorporation of different flavors [14,15]. Moreover, a survey conducted on children of age group 5–12 years showed that children have a preference for red color, sweet flavors, and fruity smell in their toothpastes [16].

Melanocytes are unique cells present in the basal and suprabasal layers of gingiva tissue that produce the pigment melanin in distinct organelles known as melanosomes through a series of intricate steps [17,18]. Typically, there are ten keratinocytes to receive the melanosomes that are extruded by the melanocyte cytoskeleton through dendrites for each melanocyte [19]. By virtue of their melanin pigment which has antimicrobial properties [20] and the control of immune function [21], melanocytes in the oral cavity contribute to the homeostasis of the gingival epithelium. The pigment melanin appears as early as 3 h after birth [22]. Due to their smaller keratinocytes and thinner epidermis, children have a lower degree of melanin pigmentation than adults. The stratum corneum’s water content rises concurrently, which lessens the requirement for the keratinocytes to make melanin pigments [23,24].

A previous in vivo study [25] showed that the concurrent use of toothpaste containing fluoride and fluoride mouthwash did not result in any cytotoxic or genotoxic effects on buccal mucosal cells. Another in vivo study further substantiated that the use of fluoride-containing toothpastes did not induce cytotoxicity or genotoxicity in buccal epithelial cells; however, sodium lauryl sulfate (SLS)-containing toothpastes resulted in cytogenetic damage [26]. Elsewhere, another in vivo study reported the irritation effects of SLS detergent in contrast to CAPB detergent on oral mucosa [27]. Several clinical studies have demonstrated that SLS results in irritation, burning sensation in the mouth, recurrent aphthous ulceration, and epithelial desquamation [28,29,30,31]. Previous studies have reported cytotoxic effects of children’s mouthrinses on various oral cells [32,33,34]. Other studies examined children’s toothpastes for cytocompatibility in L929 mouse fibroblasts [35], HSC-2 oral squamous carcinoma cells [35], human gingival fibroblasts [35,36], gingival epithelial cells [37], gingival stem cells and periodontal ligament stem cells [38]. However, it is crucial to evaluate the potential cytotoxic effects of children’s mouthwashes and toothpastes on oral melanocytes in order to identify any adverse consequences, especially as these cells form a symbiotic unit with gingival keratinocytes to export melanin pigment, which provides biological benefits of scavenging inflammation and regulating the immune response. To the best of our knowledge, the cytotoxicity of children’s toothpastes or mouthrinses on oral melanocytes has not been addressed to date. Due to their comparable histology and ultrastructure, epidermal melanocytes constitute a valuable alternative [39,40] and can be utilized to provide insights regarding the impact of mouthrinse and toothpaste exposure on melanocytes of the oral cavity.

Herein, six commercially available children’s mouthrinses and four children’s toothpastes were assessed for any potential cytotoxic effects using human epidermal melanocytes derived from lightly pigmented neonatal foreskin (HEMn-LP cells) as a model of primary oral melanocytes in vitro. The novel results of this study provide insight into the potential cytotoxicity of pediatric toothpastes and mouthrinses to oral melanocytes and can help to inform choices of oral care products with less adverse effects.

## 2. Materials and Methods

### 2.1. Materials

Six commercial children’s mouthrinses and four children’s toothpastes used in this study were purchased locally; the description of their compositions and manufacturer information are summarized in Table 1 and Table 2, respectively. CellTiter 96^®^ AQueous One Solution Cell Proliferation Assay (MTS) was obtained from Promega Corporation (Madison, WI, USA). SLS (Cat# 4509-10G) was purchased from Sigma-Aldrich (St. Louis, MO, USA) and cetylpyridinium chloride (CPC; Cat# TCH0078-25G) was procured from Avantor (Radnor, PA, USA). Culture medium 254 and human melanocyte growth supplement (HMGS) were acquired from Cascade Biologics (Portland, OR, USA). Hank’s buffered salt saline (HBSS), penicillin–streptomycin antibiotic mixture, and TrypLE Express enzyme (1×) were purchased from Thermo Fisher Scientific, Inc. (Waltham, MA, USA).

### 2.2. Cell Culture

Human epidermal melanocytes from a lightly pigmented neonatal donor (HEMn-LP), procured from Thermo Fisher Scientific Inc. (Cascade Biologics™) [41], were cultured using Medium 254 supplemented with 1% HMGS and 1% penicillin–streptomycin. These cells were maintained in a humidified incubator (95% air-5% CO_2_) at 37 °C and detached using TrypLE Express enzyme (1×) for use in various experiments. HEMn-LP cells used in this study are well-established cells that have been utilized in our previously published studies [42,43,44,45,46] as well as in other studies [47,48]. All the cell culture experiments were conducted in the laboratory at the Department of Biochemistry and Cell Biology, Stony Brook University.

### 2.3. Preparation of Mouthrinses and Toothpaste-Conditioned Medium (TCM)

Each mouthrinse (initially at a concentration of 100%) was subjected to dilution using a complete culture medium to obtain various dilutions of 1:2, 1:5, 1:10, 1:100, and 1:1000 (equivalent to 50%, 20%, 10%, 1%, and 0.1% *v*/*v*). The method of preparing toothpaste-conditioned medium (TCM) was similar to the method used in our previous study [49] and similar to that reported in another study [50]. Briefly, 0.5 g of each toothpaste was weighed in sterile tubes and combined with 1 mL of serum-free culture medium to yield a 50% *w*/*v* solution; this slurry was vortexed rapidly for a few minutes and then centrifuged for 30 min at 13,000 rpm. The supernatants were used as TCM and diluted using a complete culture medium to various ratios of 1:2, 1:5, 1:10, 1:50, 1:100, and 1:1000 (equivalent to 25%, 10%, 5%, 1%, 0.5%, and 0.05% *w*/*v*). For all experiments, toothpaste extracts were freshly prepared immediately before being added to cell cultures.

### 2.4. Cell Viability

Cell viability was determined using the MTS assay that is based on the principle of reduction by mitochondrial dehydrogenases of live cells to purple formazan crystals. HEMn-LP cells (1 × 10^4^ cells per well in 0.2 mL medium) were seeded onto 96-well plates and cultured for 72 h, after which fresh medium containing different dilutions of the test products were added, and the cultures were incubated for 2 min. Immediately following this, cultures were washed with warm HBSS solution, a new culture medium (with no test materials) was replaced in each well, and the plate was incubated for a recovery duration of 24 h at 37 °C in a humidified incubator with 5% CO_2_. Subsequently, the culture medium in each well was replaced by 100 µL of culture medium containing 20 µL of MTS dye solution and incubated for 90 min at 37 °C in the incubator. After this step, 100 µL from each well were aliquoted in a new 96-well plate, and the absorbance was recorded at the wavelength of 490 nm using a Versamax^®^ microplate reader. Cell viability was calculated based on absorbance measurements that were scaled to the untreated control group (set at 100%) for normalization.

### 2.5. Morphological Assessment

Next, 1 × 10^4^ HEMn-LP cells were plated in each well of a 96-well plate. After a 72 h culture duration, they were exposed to different dilutions of the products (mouthrinses and toothpastes) for 2 min. Subsequently, the cells were rinsed with warm HBSS, and the culture medium was renewed, allowing the cultures to recover for a period of 24 h. At this point, random fields in each well for each group were imaged in a phase-contrast mode in an Eclipse inverted microscope (Nikon, Tokyo, Japan) to observe the cellular morphologies.

### 2.6. Statistical Analysis

Data are presented as mean ± standard deviation (SD). The data distribution was checked for normality by the Shapiro–Wilk test, and statistically significant differences between the groups were evaluated using a one-way analysis of variance (ANOVA) with Dunnett’s multiple comparison test. Differences were considered statistically significant at *p* < 0.05. All the analyses were conducted using GraphPad Prism software version 9.4.1 (Graph Pad Software Inc., San Diego, CA, USA).

## 3. Results

### 3.1. Exposure to Mouthrinses Significantly Lowered Cell Viability

**Figure 1:**
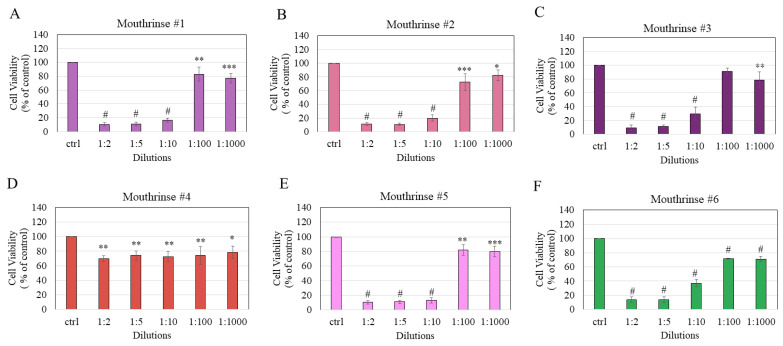
Cell viability of HEMn-LP cells estimated 24 h after a 2 min exposure to (**A**) mouthrinse #1; (**B**) mouthrinse #2; (**C**) mouthrinse #3; (**D**) mouthrinse #4; (**E**) mouthrinse #5; and (**F**) mouthrinse #6 at dilutions 1:2, 1:5, 1:10, 1:100, and 1:1000; * p < 0.05, ** p < 0.01, *** p < 0.001, and *# p <* 0.0001 compared to the control (Ctrl) group; one-way ANOVA with Dunnett’s post hoc test. All data are mean ± SD of at least three independent experiments.

The exposure of HEMn-LP cells to mouthrinse #1 resulted in significant cytotoxicity across all tested dilutions of the mouthrinse; viability was significantly lowered to 10.4% (*p* < 0.0001), 10.92% (*p* < 0.0001), 16.56% (*p* < 0.0001), 83.05% (*p* < 0.01), and 77.04% (*p* < 0.001) at dilutions of 1:2, 1:5, 1:10, 1:100, and 1:1000, respectively (Figure 1A).

The exposure of melanocytes to mouthrinse #2 induced significant decline in viabilities with residual cell viability of 11.19% (*p* < 0.0001), 10.92% (*p* < 0.0001), 19.83% (*p* < 0.0001), 72.63% (*p* < 0.001), and 82.56% (*p* < 0.05) at dilutions of 1:2, 1:5, 1:10, 1:100, and 1:1000, respectively (Figure 1B). The exposure of melanocytes to mouthrinse #3 induced significant cytotoxicity at all dilutions except the dilution 1:100; cell viability was significantly lowered to 9.61% (*p* < 0.0001), 11.35% (*p* < 0.0001), 29.76% (*p* < 0.0001), and 78.77% (*p* < 0.01) at dilutions of 1:2, 1:5, 1:10, and 1:1000, respectively (Figure 1C). Next, the results of the exposure of cells to mouthrinse #4 did not show a concentration-dependent decline in viability as the viability was significantly lowered at all dilutions in a similar manner. The residual cell viabilities were 69.87%, 74.15%, 72.69%, 74.06% (all *p* < 0.01), and 78.49% (*p* < 0.05) at dilutions 1:2, 1:5, 1:10, 1:100, and 1:1000, respectively (Figure 1D). Exposure of cells to mouthrinse #5 caused significant cytotoxicity at all tested dilutions of the mouthrinse; viability was significantly lowered to 10.34% (*p* < 0.0001), 10.99% (*p* < 0.0001), 12.97% (*p* < 0.0001), 81.95% (*p* < 0.01), and 79.92% (*p* < 0.001) at dilutions of 1:2, 1:5, 1:10, 1:100, and 1:1000, respectively (Figure 1E). Lastly, exposure of cells to mouthrinse #6 induced a significant decline in the viabilities of melanocytes at all dilutions; residual cell viability was 13.97%, 13.94%, 37%, 71.48%, and 70.88% (all *p* < 0.0001) at dilutions of 1:2, 1:5, 1:10, 1:100, and 1:1000, respectively (Figure 1F).

The mean half maximal inhibitory concentration (IC_50_) values of the cytotoxicity of mouthrinses were found to be 2.48%, 2.16%, 6.06%, 1.96%, and 6.20% (*v*/*v*) for mouthrinse #1, #2, #3, #5, and #6, respectively, while the IC_50_ values for mouthrinse #4 were not determined since it did not induce a 50% reduction at any concentration (Table 3). Based on these results, the cytotoxicity of mouthrinses follows the order #5 > #2 > #1 > #3 > #6. Collectively, the results demonstrate that mouthrinse #4 displayed the least cytotoxicity as compared to the other five mouthrinses.

### 3.2. Mouthrinses Treatment Altered Cell Morphology

**Figure 2:**
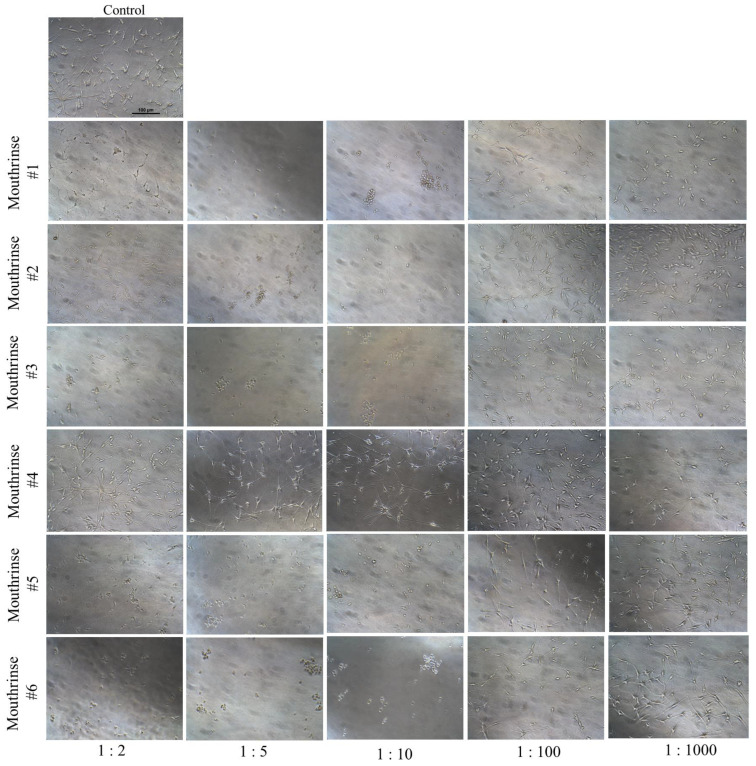
Representative phase-contrast microscopic images (×20 magnification, scale bar = 100 µm) of HEMn-LP cells taken 24 h after a 2 min exposure to the culture medium (control) and conditioned medium prepared with different dilutions (1:2, 1:5, 1:10, 1:10, and 1:1000) of children mouthrinses #1, #2, #3, #4, #5, and #6.

The morphological examination of melanocytes after exposure to the six mouthrinses showed that, except for mouthrinse #4, all the other five mouthrinses showed dramatic cell damage at the 1:2 dilution (Figure 2). Strikingly, at the dilution of 1:2, exposure to mouthrinse #3, #5, and #6 showed rounded cell bodies with no dendrites, while mouthrinse #1 and #2 showed a different morphology where cells displayed a shrunken appearance with their dendritic structure still intact (Figure 2). However, at lower concentrations (dilutions of 1:5 and 1:10), all five mouthrinses (except mouthrinse #4 which did not alter cellular morphology or cause marked cytotoxicity) caused rounded or apoptotic cell bodies with significantly fewer cells attached to the plate (Figure 2). It is worth noting that when cells were exposed to mouthrinse #3 at a dilution of 1:100, there was no discernible difference compared to the control group. Similarly, when cells were exposed to mouthrinse #4, there were no observable changes in cell morphology or the occurrence of dead or rounded cells at any dilution. These findings align with the quantitative data obtained earlier from the MTS assay, which assessed cell viability for mouthrinse #5 and #6.

### 3.3. Toothpaste Treatment Lowered Cell Viability

**Figure 3:**
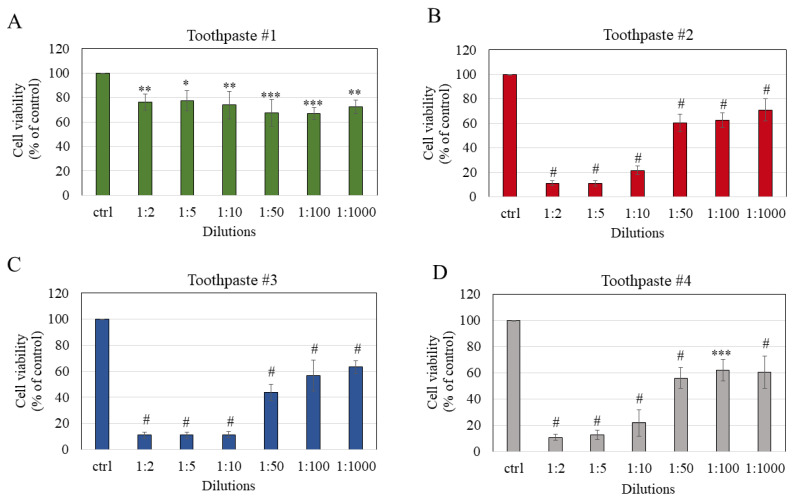
Cell viability of HEMn-LP cells estimated 24 h after a 2 min exposure to the conditioned medium of (**A**) toothpaste #1; (**B**) toothpaste #2; (**C**) toothpaste #3; and (**D**) toothpaste #4 at dilutions (1:2, 1:5, 1:10, 1:50, 1:100, and 1:1000); * *p* < 0.05, ** *p* < 0.01, *** *p* < 0.001, and # *p* < 0.0001 compared to the control group; one-way ANOVA with Dunnett’s post hoc test. All data are mean ± SD of at least three independent experiments.

The viability of melanocytes was significantly diminished after exposure to mouthrinse #1 across all tested dilutions although there was no concentration–response effect; the residual viability was 76.37% (*p* < 0.01), 77.36% (*p* < 0.05), 73.83% (*p* < 0.01), 67.71% (*p* < 0.001), 66.98% (*p* < 0.001), and 72.52% (*p* < 0.01) at dilutions of 1:2, 1:5, 1:10, 1:50, 1:100, and 1:1000, respectively (Figure 3A). Next, the exposure of cells to toothpaste #2 induced significant cytotoxicity across all tested dilutions (*p* < 0.0001 for all); viability was significantly lowered to 11.08%, 11.22%, 21.65%, 60.41%, 62.62%, and 70.96% at dilutions of 1:2, 1:5, 1:10, 1:50, 1:100, and 1:1000, respectively (Figure 3B). Cell viabilities after exposure to toothpaste #3 were significantly lowered (*p* < 0.0001 for all dilutions) to 11.15%, 11.17%, 11.40%, 43.88%, 56.66%, and 63.27% at dilutions of 1:2, 1:5, 1:10, 1:50, 1:100, and 1:1000, respectively (Figure 3C). Lastly, the exposure of melanocytes to toothpaste #4 significantly lowered viability to 10.92% (*p* < 0.0001), 12.81% (*p* < 0.0001), 21.90% (*p* < 0.0001), 56.05% (*p* < 0.0001), 61.82% (*p* < 0.001), and 60.30% (*p* < 0.0001) at dilutions 1:2, 1:5, 1:10, 1:50, 1:100, and 1:1000, respectively (Figure 3D).

The mean IC_50_ values of the cytotoxicity of toothpastes were found to be 1.25%, 0.62%, and 1.6% for toothpastes #2, #3, and #4, respectively, while the IC_50_ values for toothpaste #1 were not determined as it did not cause a decline of 50% at any concentration (Table 4). Based on these results, the cytotoxicity of toothpastes follows the order: #3 >> #2 > #4.

Taken together, the results demonstrate that toothpaste #1 displayed the least cytotoxicity as compared to the other three toothpastes, and that toothpaste #4 showed a comparable cytotoxicity profile to that of toothpaste #2.

### 3.4. Toothpastes Treatment Altered Cell Morphology

**Figure 4:**
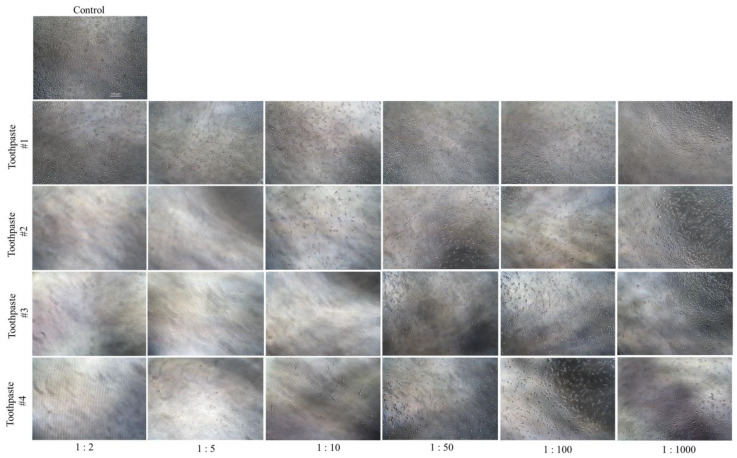
Representative phase-contrast microscopic images (×10 magnification, scale bar = 100 µm) of HEMn-LP cells taken 24 h after a 2 min exposure to the culture medium (control) and conditioned medium prepared with different dilutions (1:2, 1:5, 1:10, 1:50, 1:100, and 1:1000) of children toothpastes #1, #2, #3, and #4.

The morphological examination of HEMn-LP cells exposed to toothpaste #2, #3, and #4 showed dramatic cell loss with no visible cells adherent on the culture plate at 1:2 dilution. In contrast, toothpaste #1 had no visible cell loss with a considerable number of cells intact with no changes in morphology at 1:2 dilution (Figure 4). Interestingly, at the dilution of 1:10, exposure to toothpaste #3 continued to show cell loss with a lack of visible cells attached to the plate. In contrast, toothpaste #4 showed a lesser amount of cell loss as compared to toothpaste #3, while toothpaste #1 did not show much difference at any dilution (Figure 4). Intriguingly, when melanocytes were exposed to toothpaste #2 at a dilution of 1:10, it was observed that the cell bodies remained intact, albeit with a shrunken dendritic structure (Figure 4). The exposure of cells to all toothpastes at lower concentrations (dilutions of 1:1000 and 1:100) did not lead to any visible alterations in melanocyte dendritic morphology.

Collectively, toothpaste #1 does not lead to any morphological alterations in melanocytes, while toothpaste #3 induced the greatest morphological damage in the dilution range 1:10, 1:5, and 1:2. Results of morphological damage by toothpaste #3 align with the greatest cytotoxicity obtained earlier where it demonstrated the lowest IC_50_.

### 3.5. CPC and SLS Treatment Lowered Cell Viability

**Figure 5:**
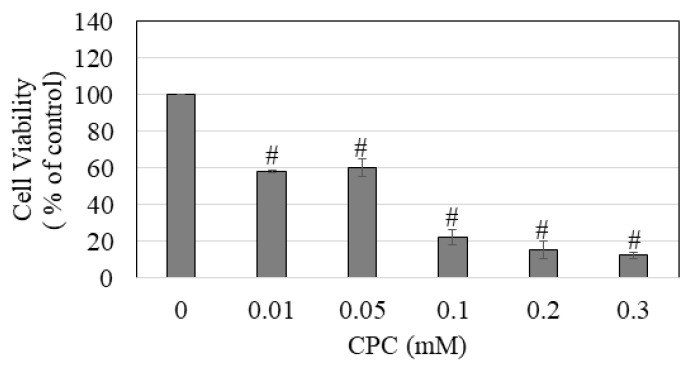
Viability of HEMn-LP cells estimated 24 h after a 2 min exposure to different concentrations (0.01–3 mM) of cetylpyridinium chloride (CPC); # *p <* 0.0001 compared to the control (CPC: 0 mM) by one-way ANOVA with Dunnett’s post hoc test. Data are an average of at least three independent experiments.

Treatment with CPC induced a concentration-dependent decline in the viability of melanocytes; viability was significantly diminished to 58%, 60.5%, 22.21%, 15.63%, and 12.42% at CPC concentrations of 0.01, 0.05, 0.1, 0.2, and 0.3 mM, respectively (Figure 5). The IC_50_ value of the cytotoxicity of CPC to melanocytes was determined to be 54.33 ± 12.38 µM.

**Figure 6:**
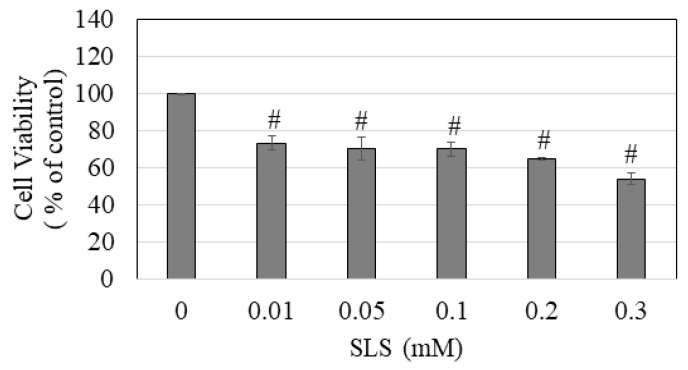
Viability of HEMn-LP cells estimated 24 h after a 2 min exposure to different concentrations (0.01–3 mM) of sodium lauryl sulfate (SLS); # *p <* 0.0001 compared to the control (SLS: 0 mM) by one-way ANOVA with Dunnett’s post hoc test. Data are an average of three independent experiments.

Next, treatment with SLS also induced a concentration-dependent decline in the viability of melanocytes; viability was significantly diminished to 73.27%, 70.31%, 70.12%, 64.77%, and 54.04% at concentrations of 0.01, 0.05, 0.1, 0.2, and 0.3 mM, respectively (Figure 6). The IC_50_ value for the cytotoxicity of SLS on melanocytes was determined to be 317.73 ± 18.89 µM, which exhibited a 5.85-fold increase compared to CPC. These results suggest that CPC exhibits much greater cytotoxicity to melanocytes than SLS.

## 4. Discussion

A clinical study [51] reported a significant decrease in melanocyte count in severe oral epithelial dysplasia; the potential cause of this phenomenon was attributed to chronic irritation caused by chemical products, resulting in melanocyte death. Irritants such as SLS, cocamidopropyl betaine (CAPB), or other detergents present in toothpastes and mouthwashes have also been implicated in the pathogenesis of oral melanoacanthoma [52]. Based on the current body of scientific literature, it is important to note that there is a dearth of in vivo investigations that have explicitly elucidated the impact of toothpaste or mouthrinse product on oral melanocytes through histological or ultrastructural examination, making a direct comparison to our study difficult. Previous studies [35,53,54,55,56,57] investigating toothpastes or mouthrinses have utilized direct incubations of test materials with gingival fibroblast cells, despite the fact that these cells are physically situated underneath the keratinocytes and melanocytes. In our study, which employed a similar direct incubation of melanocytes with products, it was hypothesized that soluble components released from toothpastes or mouthrinses during their application would penetrate the gingival epithelium and primarily affect the underlying basal layer, which predominantly comprises melanocytes that exist in smaller proportions with a ratio of one melanocyte for ~15 keratinocytes [58] and have an increased susceptibility to exposure. This exposure is anticipated to be further heightened in instances where there is abrasion or damage in the oral cavity, leading to a weakening of epithelial junctions and subsequent access of substances to melanocytes. This is especially likely in children, as their gingival barrier has lower keratinization compared to adults, and they are highly susceptible to SLS-induced barrier disruption [35,54]. Furthermore, the soluble components of these products can also contact melanocytes by systemic means through the blood.

The primary goal of this research was to examine the effects of children’s mouthrinses (#1–6) and toothpastes (#1–4) and their key surfactants, CPC and SLS, on normal human melanocytes. Toothpaste stimulation was 2 min to match the typical brushing time [59,60]. Because prior studies [56,61,62,63] on mouthrinses utilized 2 min, both mouthrinses and toothpastes had a 2 min treatment duration and a 24 h recovery period. The tetrazolium-based MTT/MTS assay has been shown to be a viable in vitro method for evaluating dental materials’ cytotoxicity [64]. We used it to examine melanocyte viability after exposure to various pediatric oral care products. Multiple studies have used MTT [34,53,54,65,66] or MTS [49,67] assays to test toothpaste or mouthwash cellular viability. MTS dissolves directly in the cell culture medium, eliminating the need for solubilization in the MTT assay [68]. We did not further use cytotoxicity assays such as the lactase dehydrogenase (LDH) membrane integrity assay due to their limited sensitivity [69] and susceptibility to detergents like SLS, which deactivate the LDH enzyme [70]. Moreover, the short half-life of the LDH enzyme (~9 h) reduces the accuracy of LDH testing in long-term cytotoxicity investigations [71]. Stannous fluoride (SnF_2_) was shown to induce greater cytotoxicity than sodium fluoride (NaF) against mouse 3T3 fibroblasts and embryonic stem cells [72]. In our study, mouthrinses #1 and #2 are comparable except that #1 contains 0.02% SnF_2_ and #2 has 0.02% NaF. Interestingly, these substances have similar melanocyte cytotoxicity patterns. Therefore, it is possible that SnF_2_ may not have greater melanocytotoxicity than NaF. It should be noted that both toothpastes #1 and #2 were fluoride free, although toothpaste #2 contained SLS which was absent in toothpaste #1. As toothpaste #2 exerted high cytotoxicity (IC_50_: 1.25%), it seems likely that SLS is the driver of cytotoxicity. Moreover, toothpaste #3 that contained both SLS and 1100 ppm fluoride (IC_50_: 0.62%) exhibited a higher level of cytotoxicity compared to toothpaste #2 which suggests that the presence of fluoride augmented the cytotoxic action. Nevertheless, toothpaste #4 (IC_50_: 1.60%), which also contains both SLS and 1100 ppm fluoride, exhibited a 2.58-fold decrease in cytotoxicity compared to toothpaste #3. This observation provides clear evidence that other components may play a role in the observed cytotoxicity and suggests that fluoride does not potentiate the cytotoxic effects. The IC_50_ values for human gingival fibroblasts exposed to NaF for durations of 1 min and 15 min were found to be 1000 ppm and 400 ppm, respectively [34]. In our previous study [43], we also showed that the cytotoxicity of NaF towards HEMn-LP cells increased with time. Specifically, an IC_50_ value of 3.09 mM (58.71 ppm fluoride) was observed after a 24 h exposure, but a lower IC_50_ value of 1.68 mM (31.92 ppm fluoride) was observed after a 72 h exposure. Nevertheless, a direct comparison between the fluoride cytotoxicity findings of our prior work and current study is not feasible for two primary reasons: (i) in this study, a 2 min exposure followed by a 24 h recovery period was employed, whereas in our previous study, cells were exposed continuously for 24 h and 72 h; and (ii) in our prior study, fluoride was examined in its isolated form as NaF, whereas in this study, toothpaste/mouthrinse formulations containing NaF along with other components were tested. Because the current study did not include a comparable treatment of a 2 min exposure with a subsequent 24 h recovery period for NaF, the accurate assessment of the role of NaF in the observed cytotoxicity cannot be made. Furthermore, it has been demonstrated that the fluoride concentration of commercial oral care products may vary from the value indicated on their labels [15,73], thereby posing a challenge in drawing definitive conclusions.

For its foaming qualities and capacity to emulsify and dissolve plaque deposits, SLS is a commonly used anionic surfactant in commercial toothpastes in concentrations ranging from 0.5% to 2% [74,75]. SLS denatures proteins and causes disruption of the phospholipid bilayers of cell membranes [76,77], and has been reported to cause adverse effects on cells of the oral cavity [34]. A 2 min exposure of human keratinocytes to SLS compromised cell viability [78]. Additionally, SLS was shown to induce skin irritation and melanocytotoxicity [79]. SLS alone or when present in children’s toothpastes altered the expression of genes linked with odontogenesis and perturbed the oxidant–antioxidant balance in zebrafish embryos [80]. Moreover, another detergent CAPB was shown to demonstrate similar adverse effects as that of SLS in the previous study [80]; the authors further showed that children’s toothpastes without any detergent were deemed the safest alternative. As the gingiva of children has less keratinization than adults, it is more susceptible to surfactant-induced injuries, particularly SLS [35,54]. In our experiments, toothpaste #1 was the only toothpaste that was recommended for use by the youngest children between the ages of 3 and 24 months, while the other three toothpastes (#2, #3, and #4) were recommended for children of age 2 years or older. Hence, it is plausible that SLS surfactant was not incorporated in the toothpaste formulation. Manufacturers are expected to use lower quantities of surfactants in toothpastes intended for use by children under the age of six, as children detest foam in particular [81]. Furthermore, after the age of six, children with mixed dentition transition to adult toothpastes that include a greater concentration of foam-generating surfactants [81]. Our results of greater cytotoxicity of SLS-containing toothpastes are consistent with other studies that similarly showed that children’s toothpastes that contained SLS were highly cytotoxic. For example, Cvikl et al. reported that SLS-containing children’s toothpastes demonstrated greater cytotoxicity to human oral fibroblasts with IC_50_ values under 5% [35], these values were in a similar range to that obtained with SLS-containing adult toothpastes in their earlier study [54] and align with the IC_50_ values obtained in our study. Another study also reported greater cytotoxicity by SLS-containing children’s toothpastes to mouse L929 fibroblasts [82]. Accordingly, the use of alternative natural surfactants [83] or CAPB-based surfactants [35] in children’s toothpastes has been suggested. It is worth noting that all of the aforementioned studies determined cell viability immediately after a 2 min exposure. However, our experimental design differs as we incorporated a 24 h recovery period before evaluating cell viability. Notwithstanding this difference, the greater cytotoxicity of SLS-containing children’s toothpastes in our experiments indicates the potential damage-inducing effects of SLS which may not recover after 24 h. The precise determination of specific components responsible for cytotoxic effects in oral care products, such as mouthrinse and toothpaste, poses a significant challenge owing to the limited availability of data on the exact amounts of chemicals supplied by manufacturers. However, the prevailing agreement among the bulk of scientific studies affirms that toothpastes containing SLS have cytotoxic properties when compared to SLS-free toothpastes.

Children often fail to adhere to the suggested practice of using toothpaste in pea-sized amounts, and this may be attributed, in part, to the monitoring of their mothers who oversee their children’s brushing but lack knowledge about the appropriate quantity of toothpaste [84]. Moreover, most children typically do not expectorate completely after brushing and rinsing [85] which results in increased ingestion of toothpastes. This was further supported by a previous study that found that children between the ages of 20 and 30 months who did not expectorate, ingested more children’s toothpaste than regular toothpaste [86]. In accordance with the guidelines provided by the National Health Service (NHS), it is recommended that children refrain from rinsing their mouths immediately after brushing with toothpaste [87]. Because young children lack sufficient control over their swallowing reflexes [88,89], about 83% of children between the ages of 3–5 had a propensity to ingest toothpaste on a regular basis [90]. Another study [91] has demonstrated that children aged 2–4 years and 5–7 years swallowed 34% and 13% of the toothpaste, respectively. Based on these reports, young children are more likely to be exposed to toothpaste components that might lead to cellular toxicity.

Mouthrinses can penetrate intraoral tissues efficiently [92]. CPC is a quaternary ammonium compound (QAC) that exhibits antiplaque and antimicrobial properties due to its cationic component, allowing it to bind negatively charged intraoral proteins, making it an efficient substitute for chlorhexidine [93,94,95]. Moreover, compared to chlorhexidine mouthrinses that are known to cause extrinsic tooth staining [96], the use of CPC-containing mouthrinses causes lesser staining [97]. In addition, CPC’s surfactant feature allows it to be equally dispersed on uneven surfaces, providing an additional contribution to antibacterial activity [98]. The effective concentration of CPC in mouthwash ranges from 0.05% to 0.1%, with 0.07% CPC being the most frequent [99,100]. Several reports have shown that CPC caused pulmonary toxicity [101], dermal toxicity [102], cardiotoxicity [103], and induced oxidative stress [103,104]. The IC_50_ values of CPC in A549 cells, a lung cell line, was shown to be 17 µM [101], while another study showed that CPC caused cardiotoxicity in zebrafish larvae at a concentration of 1.17 µM, accompanied by enhanced production of reactive oxygen species, superoxide dismutase, and glutathione [103]. Another study showed that CPC led to irritation and cytotoxicity in skin tissue and in vivo models [102].

Our results of cytotoxicity by CPC containing mouthrinse and that of pure CPC as assessed by MTS assay that evaluates mitochondrial function indicates that compromised viability might be due to the inhibitory effects of CPC on mitochondrial complex I and mitochondrial O_2_ consumption as shown previously [105,106]. An earlier report also showed that a 2 min exposure to CPC containing mouthrinse induced cytotoxicity to an L929 fibroblast cell line [107]. The immediate examination of mouthrinse cytotoxicity after a 2 min exposure was not conducted in our study. This decision was based on previous research [62] which indicated that a 0.12% CHX mouthrinse exhibited higher levels of cytotoxicity after a 24 h recovery period, as opposed to the cytotoxicity seen immediately after a 2 min exposure, in odontoblast-like cells. In order to investigate the impact of mouthrinse in conditions that closely resemble real-world use, we implemented a 24 h recovery period subsequent to a 2 min mouthrinse exposure. This experimental design enabled us to assess the effects of mouthrinse in a manner consistent with its intended use, whereby the rinse is typically expelled from the mouth after a short duration of 2 min and is similar to that employed in multiple previous studies [56,61,62,63]. The mouthrinse #4 lacks CPC, but instead incorporates a variety of natural herb-based components and essential oil extracts. Our study has demonstrated that this particular mouthrinse exhibits lower cytotoxicity compared to mouthrinses containing CPC. These results exhibit similarities to the results reported by Song et al. [108], wherein the evaluation of Garglin^®^ mouthrinses on osteoblast precursor cells was conducted. The study demonstrated that, following a 1.5 min exposure (without any recovery period), the CPC-free mouthrinse containing essential oil extracts exhibited lower cytotoxicity compared to the CPC-containing mouthrinse with a similar composition. These results suggest that CPC plays a pivotal role in the induction of the observed cytotoxic effects. Additionally, the authors compared regular and children’s brands of Garglin^®^ mouthrinses, both of which contained CPC as the active ingredient, and showed that children mouthrinses were similarly cytotoxic as regular mouthrinses. Despite exhibiting lower cytotoxicity compared to the remaining five mouthrinses, mouthrinse #4 resulted in a decline in cell viability by 20–30%. Based on the findings of this study, it can be inferred that the use of all mouthrinses is not advisable due to their cytotoxic effects after being rinsed off the tooth surface.

According to the manufacturer, the mouthrinses that were selected in this study are recommended for children aged 6 years and above. In the case of toothpastes, the age recommendations as provided by the manufacturer are 3–24 months (toothpaste #1), <2 years, 2+ years, 6+ years (both toothpastes #2 and #3), and 3+ years (toothpaste #4). Accordingly, a child below the age of 24 months has the option to use any of the 4 toothpastes, namely toothpaste #1, toothpaste #2, toothpaste #3, or toothpaste #4. Conversely, once the child reaches the age of 2 years, they may transition to using any of the 3 toothpastes, namely toothpaste #2, toothpaste #3, or toothpaste #4. Based on our findings, toothpastes #2, #3, and #4 are highly cytotoxic, rendering them unsuitable. However, toothpaste #1 was safer but still significantly decreased cell viability across all concentrations. Based on the observations made in this study, which utilized a 2 min exposure duration, it is reasonable to hypothesize that the cytotoxic reactions towards melanocytes could potentially be intensified when toothpastes are ingested by children or come into prolonged contact with the oral cavity. This could occur, for instance, due to the presence of residual soluble constituents from toothpastes in the oral cavity.

The rationale for the choice of the cell source, which has been used for this in vitro study, was predicated upon a multitude of factors. Both human skin and oral melanocytes share embryonic origins of being derived from the neural crest. Skin melanocytes share morphology, ultrastructure, and histology similar to oral melanocytes [39,40,109]. In addition, they exhibit melanocyte antigenic markers S100 and HMB-45, which are also expressed by oral melanocytes [110]. When exposed to chemical triggers like tobacco smoke or UV irradiation, the skin melanocytes are activated to increase melanin production [111]. Similarly, it has been shown that oral melanocytes can also react to tobacco smoke by inducing their pigmentation, resulting in the development of smoker’s melanosis as seen in clinical studies [112]. Skin pigmentation in children has been shown to correlate with their oral pigmentation. Specifically, children with darker skin also tend to have gingival pigmentation [113]. The melanocyte numbers are similar in the epidermis and the oral mucosa [114]. However, their melanogenic activity differs, with oral melanocytes usually exhibiting lower activity than skin melanocytes. Hence, we chose a newborn donor of Caucasian ethnicity with light pigmentation as the model to mimic oral melanocytes. This choice was made because it more accurately represents the decreased melanogenic activity of oral melanocytes under normal physiological settings. It is important to mention that oral melanocytes only become active and enhance their melanin synthesis in cases of injury, inflammation, or exposure to toxic substances. This condition has been clinically seen, particularly in situations where children develop pigmentation in their gingiva after being exposed to tobacco smoke from their parents [115,116]. We chose neonatal instead of adult skin melanocytes as under normal conditions, owing to the lack of any history of exposure to chemical triggers or oral pathology, neonatal melanocytes have lower pigmentation [23,24] and lower dendricity than adult melanocytes [117], although melanocyte frequencies in neonatal and adult skin are similar [118]. Additionally, neonatal cells have the advantage of being able to be grown and replicated to higher passages in comparison to adult melanocytes [119]. This characteristic makes neonatal cells a viable choice for conducting multiple experiments. The detergent SLS in toothpastes induced comparable cytotoxic effects in primary human keratinocytes from the skin and oral mucosa, suggesting that skin cells could be used as a substitute for oral cells [120]. Furthermore, the use of immortalized human skin keratinocytes (HaCaT cells) or mouse skin fibroblasts (L929 cells) has been common in previous studies that examined toothpastes [82], mouthwashes [107,121], or dental materials [122,123,124,125,126]. Based on these justifications, it can be inferred that utilizing skin melanocytes obtained from a neonatal lightly pigmented donor can serve as a model for oral melanocytes of children. These cells that are easily available from a commercial vendor serve as a practical and reliable initial model for assessing the cytotoxicity of different pediatric oral care products. We acknowledge that the use of melanocytes isolated from oral tissue would have yielded a culture condition that is more analogous to in vivo exposures. Only one study [127] has documented the isolation of melanocytes from oral mucosal tissues in canines. However, the authors encountered the challenge of contamination with keratinocytes during the isolation process [127]. The successful isolation of a pure culture of primary melanocytes from oral mucosa may pose a significant challenge due to the prevalent presence of keratinocytes and fibroblasts, which are more abundant in both skin and mucosa and exhibit a considerably higher rate of proliferation [128]. Currently, due to some constraints, we are unable to conduct studies on the isolation and validation of oral melanocytes. Therefore, future studies might be conducted to systematically document the isolation and validation of human oral melanocytes, as well as their assessment in conjunction with oral care products, in order to corroborate and expand upon the outcomes of our research.

This study is not without limitations. First, the investigation into the cytotoxicity induced by toothpastes and mouthrinses did not encompass an analysis of the specific mechanism of cell death, namely apoptosis, necrosis, or late apoptosis. The study conducted by Birant et al. [37] involved the investigation of the effects of children’s toothpastes on gingival epithelial cells. The authors employed flow cytometry to analyze the mechanisms underlying cell death in this context. Future investigations delving into the mechanisms underlying cell death induced by toothpastes and mouthrinses in neonatal melanocytes, as exemplified by the previous study [37], would be of interest. Second, the investigation pertaining to the influence of mouthrinses and toothpastes on the biochemical processes of neonatal melanocytes, specifically in relation to melanin synthesis, tyrosinase activity, and oxidative stress, was not undertaken, as it was beyond the scope of this study. However, it is worth noting that future studies aimed at exploring these specific endpoints would undoubtedly contribute valuable insights to the field. Another limitation of this study is that only a select number of toothpastes and mouthrinses for children were examined for melanocyte cytotoxicity. For instance, there are several different children’s toothpaste formulations available on the market (e.g., with various surfactants), and different children have various brushing practices (brushing duration and load). Additional investigations are warranted to explore alternative toothpastes and mouthrinses that may incorporate different detergents, with the aim of identifying products that exhibit complete nontoxicity towards melanocytes. Another limitation is that our in vitro model may not accurately reflect the cytotoxic effects of oral care products on melanocytes in children with increased gingival pigmentation due to their ethnicity or exposure to passive smoking. The presence of melanin pigment, which has the potential to protect against cytotoxicity, introduces an additional variable that may influence cytotoxic responses [129]. Nevertheless, our previous study showed no significant difference in cytotoxicity responses of specific adult toothpastes towards pigmented and unpigmented gingival keratinocytes [49]. A follow-up investigation on the impact of toothpaste on oral melanocytes from different racial groups of children or skin melanocytes from different ethnicities would be noteworthy. Future studies that examine and compare different toothpastes or mouthwashes and their respective ingredients on human skin and oral melanocytes would significantly contribute to this field of research. Additionally, it is important to exercise caution when applying our in vitro findings to real-world clinical settings. This is because our experimental conditions do not account for factors such as saliva, bacterial flora, or pH variations commonly found in the oral microenvironment. Therefore, additional research will be necessary to evaluate the biocompatibility of children’s mouthwashes and toothpastes in the environment of the oral cavity.

## 5. Conclusions

According to the findings of this study, SLS-free toothpaste #1 displayed the lowest cytotoxicity of all the toothpastes examined. Similarly, when compared to other mouthrinses, CPC-free mouthrinse #4 showed less cytotoxicity. Therefore, toothpaste #1 and mouthrinse #4 were identified as the preferable alternatives in relation to exhibiting reduced cytotoxicity towards healthy human neonatal melanocytes. However, it is important to acknowledge that these products still resulted in a decrease in cell viability, hence they might not be safe when used. In summary, it can be concluded that commercially available children’s toothpastes and mouthrinses exhibit a varying spectrum of cytotoxicity to neonatal melanocytes.

## Figures and Tables

**Table 1:** The compositions of children’s mouthrinses used in this study.

Mouthrinse #	Mouthrinse	Distributor	Active Ingredient	Inactive Ingredients
1	Listerine, Berry Splash	Johnson & Johnson Consumer Inc. (Skillman, NJ, USA)	Stannous fluoride 0.02% (0.01% *w*/*v* fluoride ion)	Water, sorbitol, flavor, phosphoric acid, cetylpyridinium chloride, sucralose, sodium saccharin, disodium phosphate, red 33, green 3
2	Listerine, Pink Lemonade	Johnson & Johnson Consumer Inc. (Skillman, NJ, USA)	Sodium fluoride 0.02% (0.01% *w*/*v* fluoride ion)	Water, sorbitol, flavor, phosphoric acid, cetylpyridinium chloride, sucralose, sodium saccharin, disodium phosphate, red 33, red 40
3	ACT Kids, Groovy Grape	Chattem, Inc., a Sanofi Company (Chattanooga, TN, USA)	Sodium fluoride 0.02% (0.01% *w*/*v* fluoride ion)	Water, sorbitol, flavor, cetylpyridinium chloride, sucralose, disodium phosphate, red 33, polysorbate 20, poloxamer 407, propylene glycol, benzyl alcohol, blue 1, calcium disodium EDTA, sodium benzoate, sodium phosphate, potassium sorbate
4	Tom’s of Maine, Strawberry	Tom’s of Maine, Inc. (Kennebunk, ME, USA)	Sodium fluoride 0.04% (0.02% *w*/*v* fluoride ion)	Water, glycerin, *Aloe barbadensis* leaf juice, xylitol, sodium phosphate, propanediol, benzoic acid, natural flavor, phosphoric acid, menthol, *Fragaria ananassa* (strawberry) fruit juice, *Ananas sativus* (pineapple) fruit juice, *Citrus aurantium dulcis* (Orange) juice, *Citrus limon* (Lemon) juice, Mangifera indica (Mango) juice, Rebaudioside A
5	Kids’ Anticavity, Bubblegum	Target Corp. (Mpls, MN, USA)	Sodium fluoride 0.05% (0.02% *w*/*v* fluoride ion)	Water, sorbitol, flavor, cetylpyridinium chloride, disodium phosphate, red 33, polysorbate 80, poloxamer 407, propylene glycol, benzyl alcohol, calcium disodium EDTA, sodium benzoate, sodium phosphate, disodium EDTA, sodium saccharin
6	ACT Kids, Wild Watermelon	Chattem, Inc., a Sanofi Company (Chattanooga, TN, USA)	Sodium fluoride 0.05% (0.02% *w*/*v* fluoride ion)	Water, sorbitol, flavor, cetylpyridinium chloride, sucralose, disodium phosphate, yellow 5, green 3, polysorbate 20, poloxamer 407, propylene glycol, calcium disodium EDTA, sodium benzoate, sodium phosphate, potassium sorbate

**Table 2:** The compositions of children’s toothpastes used in this study.

Toothpaste #	Name	Distributor	Active Ingredient	Inactive Ingredients
1	Tom’s of Maine, Toddler Toothpaste	Tom’s of Maine Inc., Kennebunk, ME 04043, USA	Fluoride free	Water, hydrated silica, glycerin, propanediol, xylitol, benzyl alcohol, citric acid, natural flavor, carrageenan
2	Tom’s of Maine, Silly Strawberry	Tom’s of Maine Inc., Kennebunk, ME 04043, USA	Fluoride free	Water, hydrated silica, glycerin, benzyl alcohol, calcium carbonate, sodium lauryl sulfate, natural flavor, carrageenan, *Citrus limon* (lemon) juice, Mangifera indica (mango) juice, *Citrus aurantium dulcis* (orange) juice, *Ananas sativus* (pineapple) fruit juice, *Fragaria ananassa* (strawberry) fruit juice
3	Crest Mystic (3+ years), Magical Bubblegum	Procter & Gamble, Cincinnati, OH, 45202, USA	Sodium fluoride 0.243% (0.15% *w*/*v* fluoride ion)	Water, sorbitol, hydrated silica, cellulose gum, sodium lauryl sulfate, flavor, sodium saccharin, trisodium phosphate, sodium phosphate, carbomer, red 40
4	Colgate Fluoride cavity protection (kids), Bubble fruit^®^	Colgate-Palmolive Company, NY, 10022, USA	Sodium fluoride 0.24% (0.15% *w*/*v* fluoride ion)	Water, sorbitol, hydrated silica, cellulose gum, sodium lauryl sulfate, flavor, sodium saccharin, PEG-12, blue 1, yellow-10

**Table 3:** IC_50_ values for the viability of human melanocytes treated with different mouthrinses.

Mouthrinse	IC_50_ (% *v*/*v*)
#1	2.48 ± 1.04
#2	2.16 ± 1.03
#3	6.06 ± 1.97
#4	n.d.
#5	1.96 ± 0.39
#6	6.20 ± 1.11

Values are mean ± SD; n.d. = nondetectable in the range tested.

**Table 4:** IC_50_ values for the viability of human melanocytes treated with different kinds of toothpaste.

Toothpaste	IC_50_ (% *w*/*v*)
#1	n.d.
#2	1.25 ± 0.27
#3	0.62 ± 0.17
#4	1.60 ± 0.46

Values are mean ± SD; n.d. = nondetectable in the range tested.

## Data Availability

The data used in this work are available from the corresponding author upon reasonable request.
